# Construction of a Bis(benzene sulfonyl)imide-Based Single-ion Polymer Artificial Layer for a Steady Lithium Metal Anode

**DOI:** 10.3390/polym15163490

**Published:** 2023-08-21

**Authors:** Yujie Wang, Mengmeng Zhao, Yazhou Chen, Haifeng Bao, Chen Li

**Affiliations:** 1 School of Materials Science and Engineering, Wuhan Textile University, Wuhan 430200, China; wangyujie154@163.com (Y.W.); mmzhao@dicp.ac.cn (M.Z.); baohaifeng@wtu.edu.cn (H.B.); 2 State Key Laboratory for Hubei New Textile Materials and Advanced Processing Technology, Wuhan Textile University, Wuhan 430200, China; 3 Hubei Key Laboratory for New Textile Materials and Applications, Wuhan Textile University, Wuhan 430200, China

**Keywords:** lithium metal battery, lithium metal anode, polymeric artificial layer, single ion conductor, lithium dendrite

## Abstract

Dendrite growth and parasitic reactions with liquid electrolyte are the two key factors that restrict the practical application of the lithium metal anode. Herein, a bis(benzene sulfonyl)imide based single-ion polymer artificial layer for a lithium metal anode is successfully constructed, which is prepared via blending the as-prepared copolymer of lithiated 4, 4′-dicarboxyl bis(benzene sulfonyl)imide and 4,4′-diaminodiphenyl ether on the surface of lithium foil. This single-ion polymer artificial layer enables compact structure with unique continuous aggregated Li^+^ clusters, thus reducing the direct contact between lithium metal and electrolyte simultaneously, ensuring Li^+^ transport is fast and homogeneous. Based on which, the coulombic efficiency of the Li|Cu half-cell is effectively improved, and the cycle stability of the Li|Li symmetric cell can be prolonged from 160 h to 240 h. Surficial morphology and elemental valence analysis confirm that the bis(benzene sulfonyl)imide based single-ion polymer artificial layer effectively facilitates the Li^+^ uniform deposition and suppresses parasitic reactions between lithium metal anode and liquid electrolyte in the LFP|Li full-cell. This strategy provides a new perspective to achieve a steady lithium metal anode, which can be a promising candidate in practical applications.

## 1. Introduction

With the extensive application of high-energy-storage devices in human life, it is urgent to research electrode material with high-energy density to develop the next-generation of lithium secondary batteries [1]. However, traditional commercial lithium-ion batteries are limited by the theoretical specific capacity of graphite anode (372 mAh g^−1^), thus their energy density can no longer meet the needs of social development, such as electric vehicles, artificial intelligence and portable electronic devices [2]. Lithium metal, owing to its higher theoretical specific capacity (3860 mAh g^−1^) and lowest reduction potential (−3.04 V vs. SHE), was considered as one of the most potential anode materials for next generation lithium secondary batteries [3,4]. Unfortunately, the uncontrollable lithium dendrite growth, caused by the inhomogeneous deposition of Li^+^ and persistent parasitic reactions between highly reactive lithium and liquid electrolyte are the two intricate barriers that seriously restrict the practical application of lithium metal anodes (LMA) [5,6].

Tremendous effort was devoted to overcome the lithium dendrite growth and persistent parasitic reactions of LMA, mainly including the four aspects below: (1) Optimizing the liquid electrolyte formulation via additives, such as lithium difluorophosphate, [7], fluorinated ether solvent [8] or 2,2-dimethoxy-4-(trifluoromethyl)-1,3-dioxolane [9], to improve the uniformity and compactness of SEI. (2) Utilizing inorganic, polymer or organic/inorganic composite solid-state electrolytes to inhibit lithium dendrite growth. For example, PEO-based solid-state electrolyte [10], LLZO solid-state electrolyte [11] and PEO/LLZO composite solid-state electrolyte [12]. (3) Employing a 3D current collector, such as a carbon nanotube sponge [13,14], carbon nanofibers matrix [15], and ZnO-coated hierarchical porous carbon [16], to reduce the local deposition current density. (4) Fabricating a functional artificial layer to improve the interfacial stability of LMA/electrolyte [17]. For example, a PVDF functional layer [18], PEO thin film [19] and covalent organic framework [20]. Evidently, it is the crucial to regulate the homogeneous deposition of Li^+^ and prevent the direct contact of LMA and electrolyte.

Recently, construction of a robust single-ion polymeric artificial layer (SPAL) for LMA has attracted numerous attention [21]. Firstly, the fixed anions on the matrix of SPAL can form an efficiency Li^+^ transport channel and prolong the formation of the "ion depletion layer" on the surface of LMA, which is beneficial to facilitate the homogeneous deposition of Li^+^ [22]. Secondly, the compact SPAL on LMA isolates it from electrolyte, which can avoid the parasitic reactions between LMA and liquid electrolyte effectively [23]. Furthermore, this SPAL is flexible, which is able to adapt to the volume variation during charge/discharge cycles. Song et al. incipiently proved that the thin Nafion layer on the surface of LMA can improve the performance and durability of lithium metal batteries [24]. However, the low ionic conductivity of the thin Nafion layer coursed by a strong binding energy between Li^+^ and sulfonic acid group will discount the Li^+^ transport rate during the charging and discharging processes. Therefore, Weng et al. introduced POSS nano-particles into a SPEEK layer to facilitate the dissociation of Li^+^, thereby accelerating the ion transport [25]. Moreover, Jiang et al. combined the ClO_4_^−^-decorated metal-organic framework (UiO-66-ClO_4_) and flexible lithiated Nafion binder to construct biomimetic ionic channels for the artificial solid-electrolyte interfaces [26]. However, the Li^+^ conductivity of an organic single-ion polymer is intrinsically lower compared to the commercial electrolyte as reported [21]. 

**Scheme 1:**
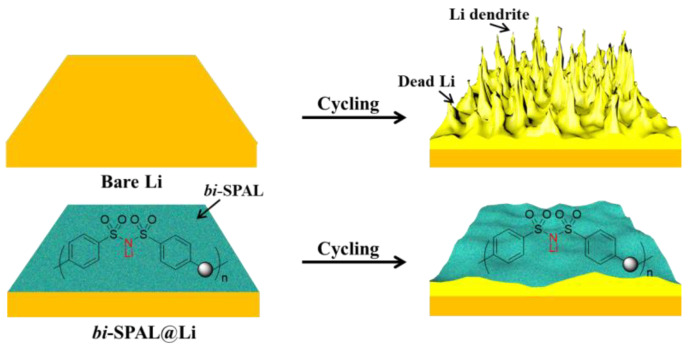
The Comparison of Li deposition behavior on the bare Li and *bi*-SPAL@Li anode.

Herein, 4, 4′-dicarboxyl bis(benzene sulfonyl)imide was copolymerized with 4,4′-diaminodiphenyl ether to obtain a single-ion conductor (LiPBIA, as shown in Scheme S1). Relying on the solution-casting method, the LiPBIA blended with PVDF was attached to a lithium foil surface to form a bis(benzene sulfonyl)imide based SPAL (*bi*-SPAL). On the one hand, the continuous aggregated Li^+^ clusters on fixed bis(benzene sulfonyl)imide anions endow prosperous Li^+^ transport channels, allowing a fast and uniform Li^+^ deposition process, which can effectively inhibit the growth of lithium dendrite. On the other hand, the *bi*-SPAL, as an interlayer between electrolyte and LMA, isolates the contact of the two, impeding the parasitic side reactions between them (Scheme 1). By employing this *bi*-SPAL on LMA, the coulombic efficiency (CE) of the Li|Cu half-cell, interfacial stability of the Li|Li symmetrical cell and cycling performance of the LFP|Li full-cell were detected systemically.

## 2. Materials and Methods

### 2.1. Materials

4-methyl benzenesulfonyl chloride (98%), 4-methyl benzenesulfonamide (98%), 4, 4′-diamino diphenyl ether (98%) and potassium permanganate were purchased from Sinopharm Chemical Reagent Co., Ltd (Shanghai, China) without further purification. Anhydrous pyridine (AR, H_2_O < 10 ppm), anhydrous dimethyl sulfoxide (AR, H_2_O < 10 ppm) and anhydrous N-methyl-2-pyrrolidone (AR, H_2_O < 10 ppm) were purchased from Aladdin Co., Ltd (Shanghai, China). Triphenyl phosphite (TPP) was purchased from Sinopharm Chemical Reagent Co., Ltd (Shanghai, China). and dried with an activated 4A molecular sieve for 7 days. Methanol (AR) was purchased from Sinopharm Chemical Reagent Co., Ltd (Shanghai, China). Poly(vinylidene fluoride) (PVDF, average Mw~400,000) was purchased from Sigma Aldrich Co., Ltd (Shanghai, China). Carbonate-based electrolyte (1 M LiPF_6_ in EC/DMC, v:v = 1:1) was purchased from DodoChem (Suzhou, China) without any additives. LiFePO_4_ with encased carbon was purchased from Tianjin STL Energy Technology Co., Ltd (Tianjin, China). Lithium foil (*Φ* 16 mm) was purchased from Hefei Kejing Co., Ltd (Hefei, China). 

### 2.2. Synthesis of Materials

**Scheme 2:**
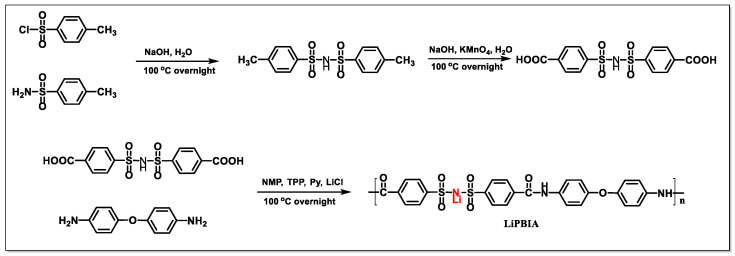
The synthesis route of LiPBIA.

Firstly, as shown in Scheme 2, 300 mL of deionized water was added into a 500 mL two-necked flask, and 0.102 mol of NaOH was slowly added under magnetic stirring. After the NaOH was completely dissolved, 0.10 mol 4-methylbenzene sulfonamide was introduced and the temperature was raised to 95 °C. Finally, 0.05 mol 4-methylbenzene sulfonyl chloride was slowly added within 2 h and the reaction was continued for 12 h at 95 °C. Then, the pH was neutralized to 7, along with a precipitated white solid. Next, the white precipitate was removed by filtering, and the pH of the filtrate was adjusted to 1, thus reaching the target product of the white solid (4, 4-dimethyl bis(benzene sulfonyl)imide, MBSI). Secondly, 0.05 mol of MBSI and 0.05 mol NaOH were added to a 500 mL two-necked flask with 200 mL deionized water. Then, KMnO_4_ was added 5 times and slowly processed under magnetic stirring for 12 h at 100 °C. After the reaction, the by-product, MnO_2_, was filtered and the pH of the filtrate was adjusted to 1 [27]. After recrystallization processing, the target product, 4, 4′-dicarboxyl bis(benzene sulfonyl)imide (CBSI), was obtained. Lastly, 0.01 mol of 4,4′-diaminobenzene diphenyl ether, 0.01 mol of CBSI, 20 mL N-methyl-2-pyrrolidone, 15 mL of Py and 5.2 mL of TPP were added in a 100 mL two-necked flask and reacted at 100 °C for 12 h under the argon flow. Then, the mixture was poured into 100 mL of cold methanol to precipitate the polymer (poly(bis(4-amino benzene) ether-alt-bis(4-carboxyl benzene sulfonyl)imide)amide), and further washed by methanol several times. The polymer was neutralized by LiOH to obtain the target product of LiPBIA.

### 2.3. Preparation of Electrodes

*Bi*-SPAL@Cu electrode: 75 mg of LiPBIA powder and 25 mg of PVDF was dissolved in 10 mL of anhydrous DMSO to form a transparent solution. The solution was casted on a flat Cu foil (5 cm × 10 cm), followed by removing the solvent through heat treatment. The *bi*-SPAL-coated Cu electrode was punched into small rounds (diameter ~ 16 mm) followed by vacuum drying.

*Bi*-SPAL@Li electrode: 75 mg of LiPBIA powder and 25 mg of PVDF was dissolved in 10 mL of anhydrous DMSO at an argon-filled glove box to form a transparent solution. Subsequently, 50 µL of the mixed solution was dropped onto the Li foil (with diameter of 16 mm) and dried at 60 °C for 12 h in the argon-filled glove box. 

LFP electrode: 140 mg of LFP powder, 40 mg of acetylene black, 20 mg of PVDF and an appropriate amount of NMP were proportionally placed in a 5 mL beaker followed by stirring for 12 h. Then, the slurry was spread on an Al foil by a doctor blade and the NMP was removed by air blowing at 80 °C for 1 h. Finally, the LFP cathode was cut into several rounds (diameter is 12 mm, active material loading is ~1.5 mg cm^−2^), which were dried at 80 °C under vacuum for 12 h. 

### 2.4. Cell Assembly and Electrochemical Test

The asymmetric Li|Cu half-cells were assembled with bare Cu foil, *bi*-SPAL@Cu foil as the working electrode, or bare Li foil as the counter electrode, and 1 M of LiPF_6_ was dissolved in the EC/DMC (v:v = 1:1) solvent as the carbonate-based electrolyte, and Celgard 2300 microporous polyolefin film was used as the separator. The cells were tested on a LAND electrochemical testing system at 25 °C. Specifically, 1 mAh cm^−2^ of Li is plated on the bare Cu or *bi*-SPAL@Cu by applying a constant current density of 1 mA cm^−2^ for 1 h and then stripped at the same current density with the opposite polarity until the voltage raised to 1.0 V. The coulombic efficiency (CE) of each cycle was calculated by dividing the Li stripping capacity with the Li plating capacity and then multiplied by 100%.

The symmetric Li|Li cells were assembled by sandwiching the Celgard 2300 microporous polyolefin film between two pieces of the bare Li or *bi*-SPAL@Li electrode and adding the carbonate-based electrolyte, same as the Li|Cu cells. The symmetric cells were cycled at capacity of 0.5 mAh cm^−2^ with a density of 1mA cm^−2^ on a LAND electrochemical testing system at 25 °C.

The LFP|Li full cells were assembled similar to the Li|Cu half-cells, where the LFP cathode replaced the Cu foil as the working electrode. Before cycling, the electrochemical impedance spectroscopy (EIS) measurements were performed on the VMP3 workstation with a frequency range from 100 kHz to 0.01 Hz with 10 mV fluctuations. The cycle performance were conducted on LAND at 25 °C at 0.2 C and 1 C within the discharge and charge cut-off voltages of 2.5 V and 4.2 V.

### 2.5. Characterization

The ionic conductivity of PP/Liquid electrolyte and *bi*-SPAL were measured with the configuration of ‘SS|film|SS’, i.e., one piece of film wetted by commercial liquid electrolyte was sandwiched between two stainless-steel current collectors. Then, an alternating current with the voltage amplitude of 5 mV from 1 MHz to 100 Hz was applied at 25 °C. In the end, the cell was disassembled in a glove box to take out the polymer electrolyte film for thickness measurement by a micrometer screw. The ionic conductivity was calculated via the Eq of σ = *l*/*RA*, where *l*, *R* and *A* stand for the film thickness, the resistance and the area of the film, respectively.

The structure of LiPBIA was characterized by an NMR spectrometer (AVANCE III HD 400 MHz, BRUKER, Karlsruhe, Germany) with DMSO-d_6_ as the solvent. The morphology of bare Li and *bi*-SPAL@Li before and after cycling, was captured by scanning electron microscopy (FE-SEM, SU8010, HITACHI, Tokyo, Japan). The surficial chemical constitution was detected by X-ray photoelectron spectroscopy on the PHI 5000 Versa system (ULVAC-PHI, Kangawa, Japan).

## 3. Results and Discussions

**Figure 1:**
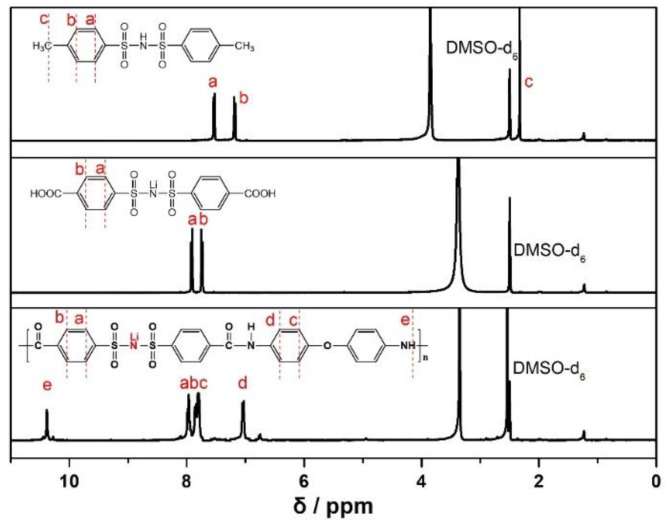
^1^H NMR characterization of MBSI, CBSI and LiPBIA.

The chemical structure of MBSI, CBSI and LiPBIA was collected by the ^1^H NMR spectra, as shown in Figure 1. For MBSI, the two double peaks at 7.54 ~ 7.52 ppm (a) and 7.19 ~ 7.17 (b) ppm were assigned to the adsorption of aromatic protons (a & b), respectively [28]. A single peak of 2.33 ppm (c) was ascribed to protons of methylene (c). In addition, the ratio of the “a”, “b” and “c” peak area closed to 2:2:3, corresponding to the structure of MBSI molecule. For CBSI, the peak of methylene protons disappeared. At the same time, the two double peaks, “a” and “b”, shifted to the lower field (7.92→7.90 ppm, 7.75→7.73 ppm), respectively, which as a result, the methyl group was oxidized to the carboxyl group. For the LiPBIA, five peaks at 10.38 (e), 7.98–7.96 (a), 7.86–7.84 (b), 7.81–7.79 (c) and 7.05–7.03 (d) were observed, respectively. The hydrogen marked with ‘a’, ‘b’, ‘c’ and ‘d’ could be denoted as ‘benzene hydrogen’, while the hydrogen labeled by ‘e’ was assigned to ‘amide hydrogen’, indicating the target product of LiPBIA was successfully synthesized.

**Figure 2:**
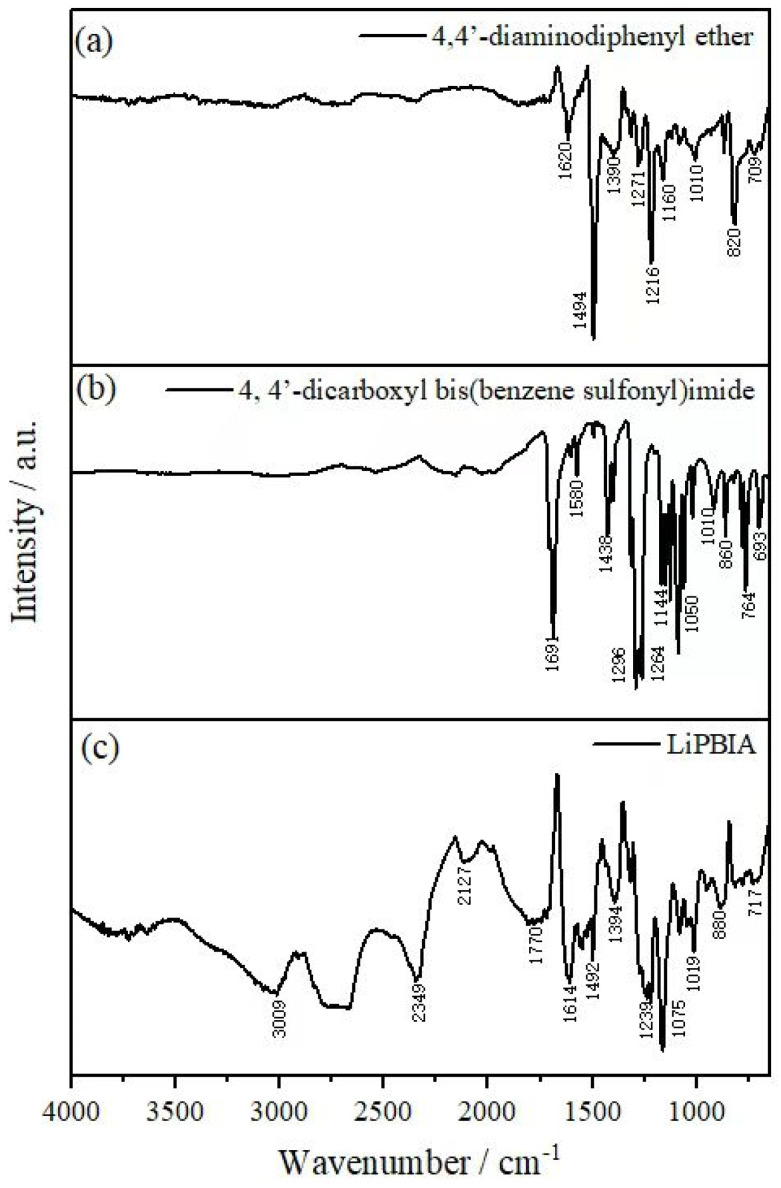
The FTIR spectra. (**a**) 4, 4′-dicarboxyl bis(benzene sulfonyl)imide. (**b**) 4,4′-diaminodiphenyl ether. (**c**) LiPBIA copolymer.

The chemical structure of LiPBIA was further collected by the FTIR spectra, as shown in Figure 2. For 4, 4′-dicarboxyl bis(benzene sulfonyl)imide (Figure 2a), the peaks of 1691 cm^−1^ and 1438 cm^−1^ are ascribed to the stretching vibration absorption peak of the carbonyl group in the carboxyl function group, which is a special function group of the structure. The peaks of 1296 cm^−1^, 1264 cm^−1^ and 1010 cm^−1^ are attributed to the bending vibrations and symmetric stretching vibrations of the -SO_2_- group and the symmetrical stretching vibrations of the S=O bond in the sulfonylimide group. The peaks of 764 cm^−1^ and 693 cm^−1^ belonged to the symmetric stretching vibrations of 1,4-substituted benzene and benzene, respectively. For 4,4′-diaminodiphenyl ether (Figure 2b), the peak of 1620 cm^−1^ belonged to the bending vibrations of the -NH- bond of the amino group, which is the function group of the 4,4′-diaminodiphenyl ether. The peaks of 1271 cm^−1^ and 1160 cm^−1^ are attributed to the stretching vibration of the Ar-O bond. The peak of 1010 cm^−1^ is attributed to the stretching vibration of the R-O bond. The peaks of 820 and 709 cm^−1^ are ascribed to the rocking vibration of the -NH- in the amino group. For the lithiated polyamide copolymer LiPBIA (Figure 2c), it is clear that the characteristic peak of the -COOH bond at 1620 cm^−1^ disappeared. The peaks of 1239 cm^−1^ and 1075 cm^−1^ belonged to the bending vibrations, symmetric stretching vibrations of the -SO_2_- bond and the symmetrical stretching vibrations of the S=O bond in the sulfonylimide group. The 717 cm^−1^ peak is attributed to the symmetric stretching vibrations of the C-H bond in the 1,4-substituted benzene. Furthermore, the peaks of 1492 cm^−1^,1394 cm^−1^ and 880 cm^−1^ bending vibrations of the -NH- bond of the secondary amine were reformed, which demonstrated that the LiPBIA were synthesized successfully. The two precursors are linked by a secondary amide, which is clearly identified in Figure 2c. The XRD results show that LiPBIA has a clear and sharp peak of 2θ = 20° (Figure S1), which indicates a relatively good crystallinity. Therefore, combining the characterization of ^1^H NMR, FTIR and XRD, can prove that the LiPBIA copolymer was successfully synthesized. In addition, the LiPBIA copolymer was soaked in the EC/DMC organic solvent for several hours through design experiments. There is no solid residue, which shows the dissolution stability (Figure S2). This phenomenon indicates that the LiPBIA copolymer is suitable as a protective layer for lithium metal batteries.

**Figure 3:**
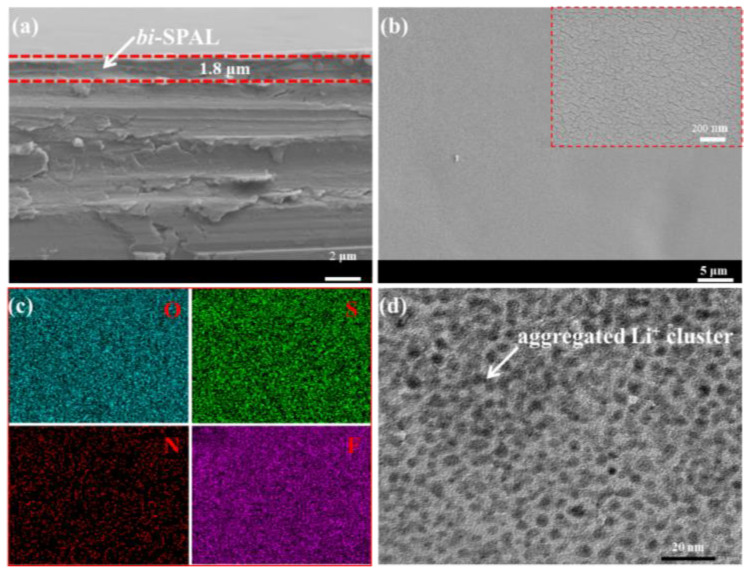
(**a**) Cross section and (**b**) surficial SEM of *bi*-SPAL@Li (high-magnification insert). (**c**) EDS mapping of the *bi*-SPAL@Li. (**d**) TEM of the *bi*-SPAL.

After confirming the structural information of LiPBIA, we tested the ionic conductivity of SPAL. It reached up to 0.96 mS cm^−2^ when soaked with liquid electrolyte (Figure S3). Such a high value is almost comparable to 1.12 mS cm^−2^ of commercial liquid electrolyte, which is attributed to the large amount of lithiated bis(benzene sulfonyl)imide anions, providing more sites for Li^+^ dissociation and transportation [29] Subsequently, a mixture of LiPBIA and PVDF (3:1) was employed to fabricate SPAL on the surface of LMA (denoted as *bi*-SPAL@Li). With the assistance of SEM, we observed that the SPAL was fitted on the surface of LMA evenly and compactly as shown in Figure 3a. Between SPAL and LMA, there was no crevice, while the thickness of the SPAL is approximately 1.8 μm. Such a compact SPAL with a certain thickness appressed on LMA can avoid the direct contact between the LMA and electrolyte effectively. Figure 3b displayed the surface section of the *bi*-SPAL@Li electrode, which demonstrated that the surface is smooth and close. Furthermore, even at a higher magnification, it still exhibits a contact and uniform morphology (insert of Figure 3b). Mapping images (Figure 3c) illustrate that the distribution of O, S, N, F elements on *bi*-SPAL @Li electrode is homogeneous, further suggesting that the SPAL is covered on the LMA continuously and densely. As zoomed in by high-resolution transmission electron microscopy (HRTEM), the typical microphase separation structure of SPAL is blindingly obvious, and tightly aggregated clusters are uniformly dispersed (Figure 3d), which formulates an efficient Li^+^ transport channel, ensuring fast and homogeneous Li^+^ transport [30]. These results suggest that SPAL has the potential to serve as an artificial SEI for LMA, in order to block the liquid electrolyte and guide the uniform deposition of Li^+^.

**Figure 4:**
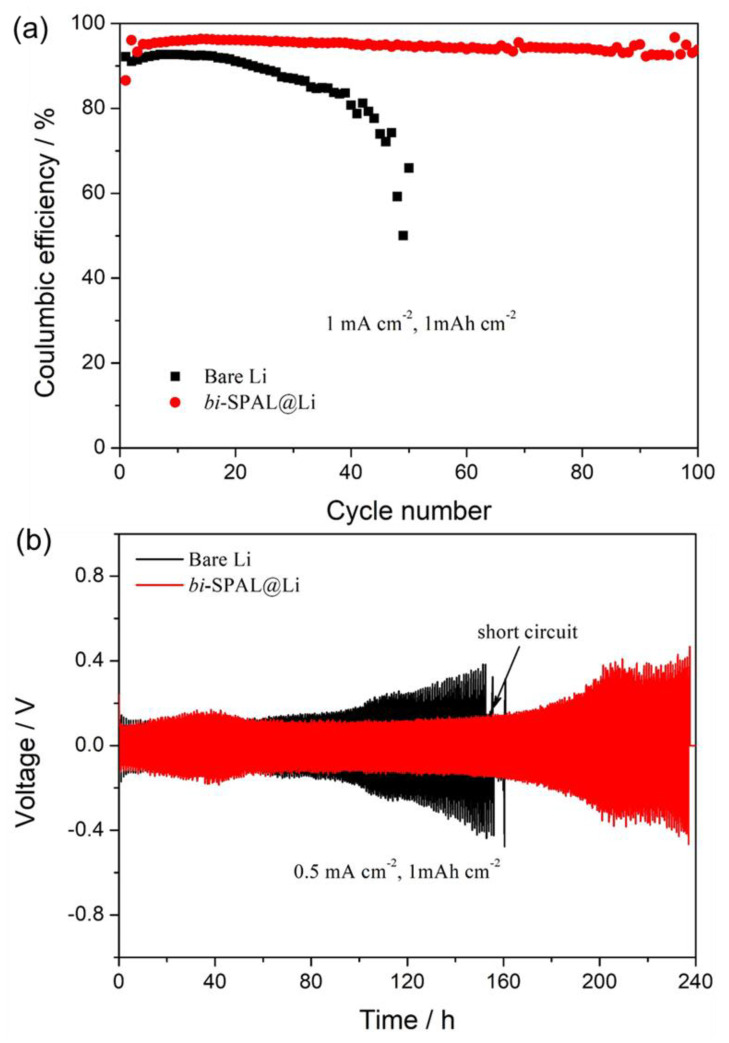
The comparison of electrochemical performance with or without *bi*-SPAL. (**a**) Coulombic efficiency of Li|Cu half-cells at 1 mA cm^−2^ (1 mAh cm^−2^). (**b**) Voltage profiles of the Li plating/stripping processfor Li symmetric cells at 0.5 mA cm^−2^ (1 mAh cm^−2^).

The CE of the Cu electrode was compared with the *bi*-SPAL@Cu electrode to investigate the influence of *bi*-SPAL to reversible Li plating/stripping performance. In the first cycle, the CE of the *bi*-SPAL@Cu electrode (~ 86.5%) was relatively lower than 92.1% of the bare Cu electrode (Figure 4a), which is attributed to the larger Li nucleation overpotential (223 mV vs. 79 mV, Figure S4a) caused by the initial unstable interface between the Cu electrode and *bi*-SPAL. After an experienced one cycle, the Cu|*bi*-SPAL interface was stabilized. The CE of the *bi*-SPAL@Cu electrode increased to 96.1% at the third cycle, rapidly, which still remained at 93.9% after 100 cycles. With regard to bare Cu electrode, the CE in the third cycle was barely attenuated, dropping to 78.1% vertiginously after 40 cycles. Meanwhile, the Li stripping/plating overpotential for *bi*-SPAL@Li was about 97 mV at the third cycle, which was maintained for 100 cycles continuously, thus, superior to the bare Cu electrode (Figure S4b–d). Furthermore, Li|Li symmetric cells were assembled to evaluate the interfacial stability of LMA/electrolyte. As indicated by the black line in Figure 4b, the overpotential of the bare Li symmetrical cell increased rapidly after 90 h. This illustrated that the bare Li anode could only work effectively in a limited time (<90 h), which owes to the complicated side reactions between the LMA and liquid electrolyte [31]. The side reactions that consumed a portion of the Li metal led to an inferior performance, meanwhile, a more complex surface appeared, which affected the Li^+^ transfer efficiency, and the generation of lithium dendrite was exacerbated [32]. At 152 h, short circuiting took place, which is attributed to the unmanageable lithium dendrite formation that connects the positive and negative electrodes together. When coating with *bi*-SPAL, the symmetrical Li|Li cell, using the *bi*-SPAL@Li electrode, displayed a much more stable Li plating/stripping behavior for 180 h and cut off at 240 h. More importantly, the polarization potential of the *bi*-SPAL@Li symmetric cell is similar to the bare one, which is attributed to the appreciable ionic conductivity of *bi*-SPAL. These results indicate that the as-prepared *bi*-SPAL is indeed critical for facilitating fast Li^+^ transport and reversible Li plating/stripping [33]. 

**Figure 5:**
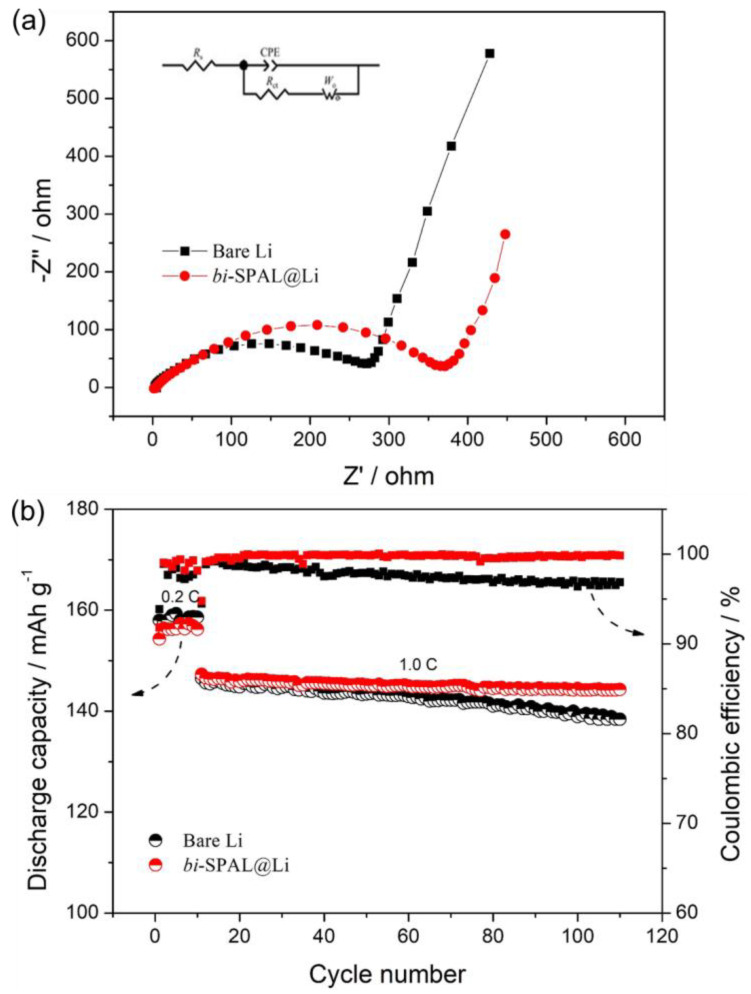
The comparison of battery performance with or without *bi*-SPAL. (**a**) Nyquist plots of the LFP|Li full cell before tested; (**b**) Cycling performance of the LFP|Li.

Ultimately, the LFP|Li full-cell was assembled and tested to further explore the practical application of the *bi*-SPAL@Li electrode with bare Li for comparison. Before the cycling test, the electrochemical impedance spectroscopy (EIS) was measured and the EIS results, with the equivalent circuit, are shown in Figure 5a. Clearly, the *Rs* (ohmic resistance) and *Rct* (resistance of charge transfer reaction between the electrolyte and the electrode) of full-cell fabrication with the *bi*-SPAL@Li electrode was 11.3 Ω and 359.2 Ω, respectively, which was relatively higher than that of the bare Li electrode (10.6 Ω, 272.5 Ω). This is ascribed to the slightly lower ionic conductivity of *bi*-SPAL compared to the liquid electrolyte (Figure S3), resulting in the increase of both *Rs* and *Rct*. Therefore, the discharge capacity of the full cell, with *bi*-SPAL@Li as the anode, was about 156.3 mAh g^−1^ at 0.2 C, slightly lower than 158.2 mAh g^−1^ of the bare Li anode (Figure 5b). Intriguingly, when the charge/discharge rate increased to 1 C, the discharge capacity of the two cells was almost equal (146 mAh g^−1^). After 100 cycles, the cell with the *bi*-SPAL@Li anode still delivered a discharge capacity of 144.5 mAh g^−1^ with CE as high as 99.84 %, which was superior to the value of 138.4 mAh g^−1^ and 96.87 % upon the bare Li. The corresponding charge/discharge curves at 1 C rates are shown in Figure S5. At the first cycle, the charge/discharge curve of both *bi*-SPAL@Li and the bare Li cell remained almost the same. However, after 100 cycles at 1 C, the charge/discharge platform of the bare Li cell was shorter than the *bi*-SPAL@Li, which is attributed to the dendrite growth and parasitic reactions on the surface of the bare Li. These results confirm that the *bi*-SPAL is beneficial for the cyclic stability of lithium metal batteries.

**Figure 6:**
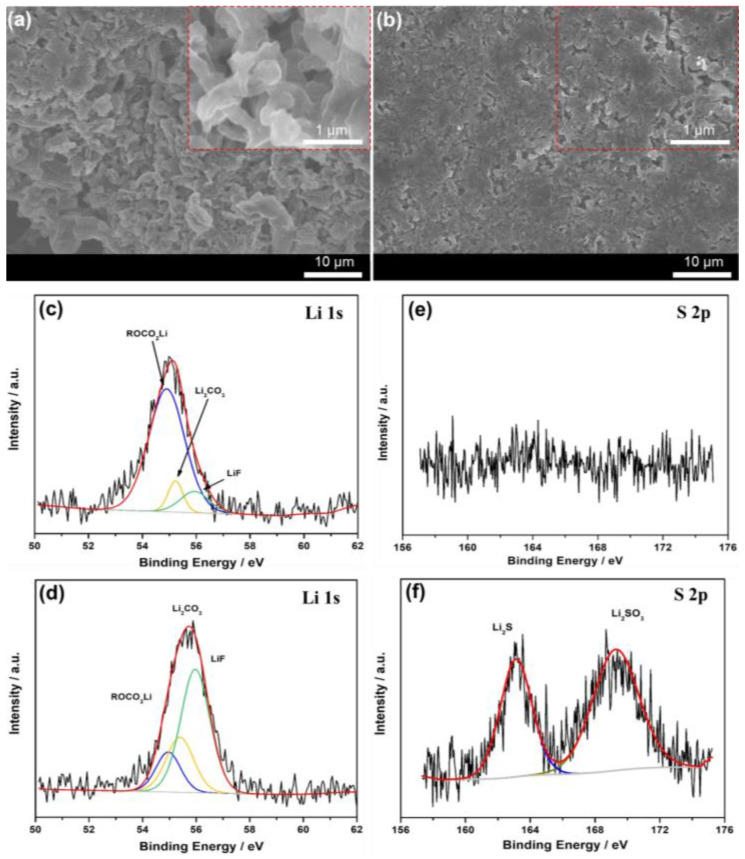
Characterization of Li foil in the LFP|Li full cell after 100 cycles at 1C. SEM images of (**a**) the Bare Li and (**b**) *bi*-SPAL@Li anode. Li 1s XPS spectra of (**c**) the Bare Li and (**d**) *bi*-SPAL@Li anode. S 2p XPS spectra of (**e**) the Bare Li and (**f**) *bi*-SPAL@Li anode.

The SEM analysis was utilized to explore the surficial morphology and elemental state of bi-SPAL after 100 cycles. As shown in Figure 6a, the surface of bare LMA turns to rather porous and numerously isolated dendritic particles, while the LMA protected by *bi*-SPAL does not change significantly (Figure 6b). The composition varieties of the cycled LMAs were further revealed by Li 1s and S 2p XPS analysis. For bare LMA (Figure 6c&e), the Li 1s XPS spectra could be fitted to the peaks of ROCO_2_Li (54.8 eV), Li_2_CO_3_ (55.3 eV), LiF (55.9 eV) and no S 2p peaks were observed. For *bi*-SPAL@Li (Figure 5d&f), in addition to the peaks of Li 1s, the peaks of S 2p also appeared, which could be fitted to Li_2_S (163.2 eV) [34], LiSO_3_ (169.4 eV), respectively. The stronger deconvoluted peak of LiF and unique peaks of S 2p are attributed to the reduction of PVDF and LiPBIA with LMA, respectively, which is beneficial to form a uniform and robust SEI layer [31]. Thus, the parasitic reactions between LMA, the liquid electrolyte and the Li dendrite growth can be inhibited. The EIS results measured after 20 cycles at 1 C further conform this phenomenon. As shown in Figure S6, the *Rct* of LFP/Li cell fabricated with the *bi*-SPAL@Li electrode was 188.9 Ω, much lower than the bare one (250.3 Ω), which is attributed to the formation of steady SEI layer for *bi*-SPAL@Li. 

## 4. Conclusions

In this work, a unique *bi*-SPAL was successively prepared. Attaching this *bi*-SPAL on lithium foil formed an even, dense and compact protective cover, able to inhibit the uncontrollable growth of lithium dendrite and impede the parasitic side reactions between LMA and the liquid electrolyte during the charge–discharge process, thus improving the interfacial stability of LMA. By inducing *bi*-SPAL@Li to the Li|LFP cell, a high discharge capacity of 144.5 mAh g^−1^ with CE at 99.84% was achieved at 1 C for 100 cycles. Surficial morphology and elemental state analysis of bi-SPAL, after battery reaction further verified the stabilization mechanism of *bi*-SPAL on LMA. 

## Data Availability

The data presented in this study are available on request from the corresponding author.

## Supplementary Materials

The following supporting information can be downloaded at: https://www.mdpi.com/article/10.3390/polym15163490/s1, Figure S1: The XRD spectra.of 4, 4′-dicarboxyl bis(benzene sulfonyl)imide, 4,4′-diaminodiphenyl ether and LiPBIA copolymer. Figure S2: The optical photograph ofthe LiPBIA dissolved in EC/DMC mixture solvent and after the EC/DMC mixture solvent evaporation. Figure 3: Ionic conductivity characterization of liquid electrolyte and *bi*-SPAL soaked liquid electrolyte. Figure S4: Plating/stripping curves of “Li|bare Cu” and “Li|*bi*-SPAL@Cu” cells at different cycles. Figure S5. The Nyquist plots of the LFP|Li full cell after 20 cycles at 1 C with or without *bi*-SPAL. Figure S6: Charge/discharge plots of LFP based lithium metal batteries at cycles.
