# Supplementary material for: Construction of a Bis(benzene sulfonyl)imide-Based Single-ion Polymer Artificial Layer for a Steady Lithium Metal Anode
Source: Polymers (Basel). 2023 Aug 21;15(16):3490. doi: 10.3390/polym15163490 (PMC10458740; doi:10.3390/polym15163490)
Supplement: Supplementary file 1 [file polymers-15-03490-s001.zip › polymers-2464425-supplementary.pdf]

Construction of A bis(benzene sulfonyl)imide based single-ion polymer artificial layer for steady lithium metal anode

Yujie Wang [1, 2, #], Mengmeng Zhao [2, #] , Yazhou Chen[1, 2, 3*], Haifeng Bao [1] and Chen Li [1,*]

1 School of Materials Science and Engineering, Wuhan Textile University, 430200 Wuhan, China. wangyujie154@163.com; yzchen@wtu.edu.cn; baohaifeng@wtu.edu.cn; cli@wtu.edu.cn.

2 State Key Laboratory for Hubei New Textile Materials and Advanced Processing Technology, Wuhan Textile University, 430200 Wuhan, China. mmzhao@dicp.ac.cn.

3 Hubei Key Laboratory for New Textile Materials and Applications, Wuhan Textile University, 430200 Wuhan, China.

* Correspondence: yzchen@wtu.edu.cn (Y. Z. C.); cli@wtu.edu.cn (C. L.)

# Both authors contributed equally.

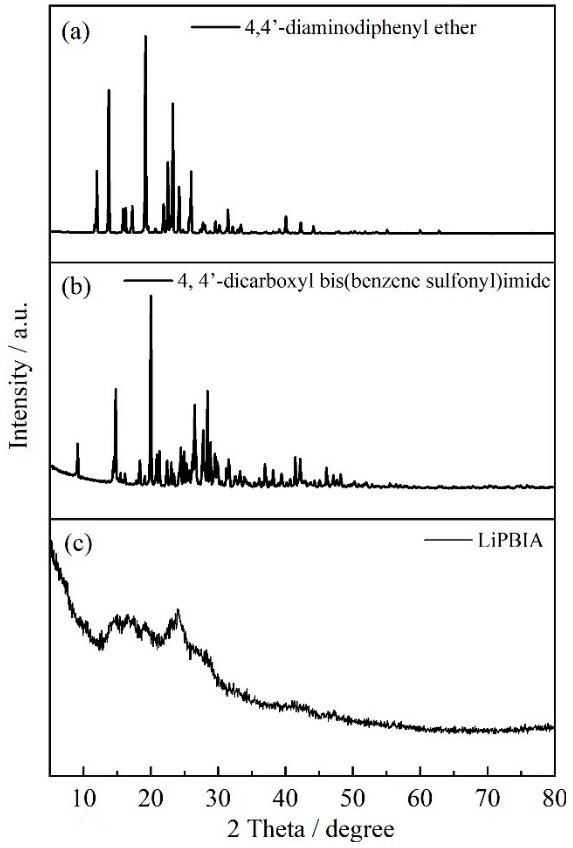

Figure S1. The XRD spectra. (a) 4, 4'-dicarboxyl bis(benzene sulfonyl)imide. (b) 4,4'-diaminodiphenyl ether. (c) LiPBIA copolymer.

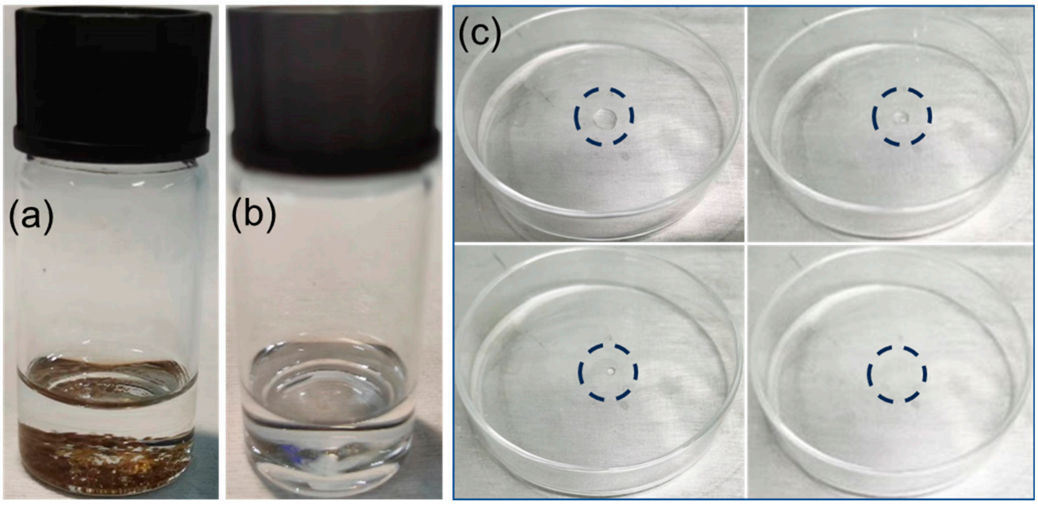

Figure S2. The optical photograph of (a) the LiPBIA dissolved in EC/DMC mixture solvent, (b) the EC/DMC mixture solvent after removed the LiPBIA particles and (c) the residual condition after the EC/DMC mixture solvent evaporation.

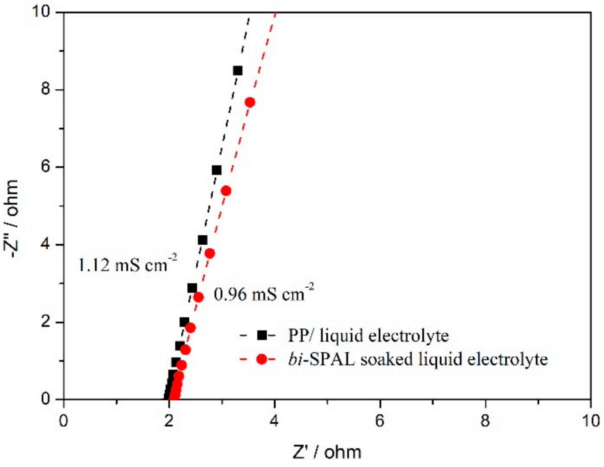

Figure S3. Ionic conductivity characterization of liquid electrolyte and *bi*-SPAL soaked liquid electrolyte.

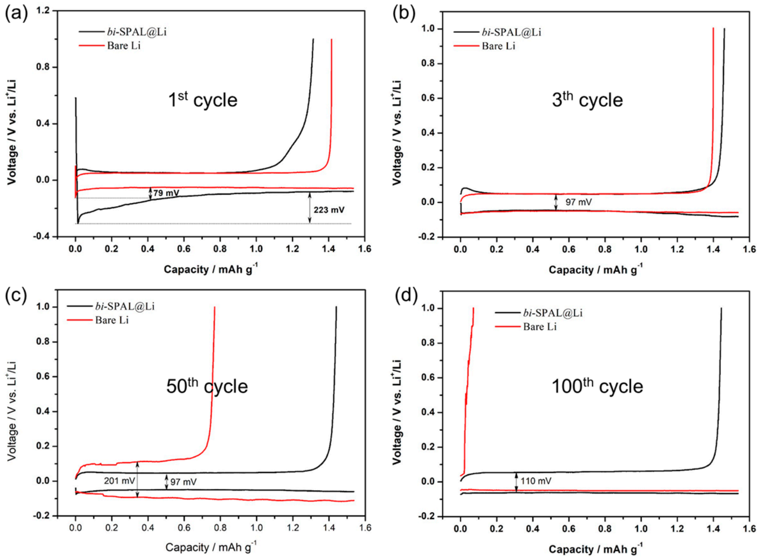

Figure S4. Plating/stripping curves of “Li | bare Cu” and “Li | *bi*-SPAL@Cu” cells. (a) The first cycle; (b) The third cycle; (c) The 50th cycle; (d) The 100th cycle.

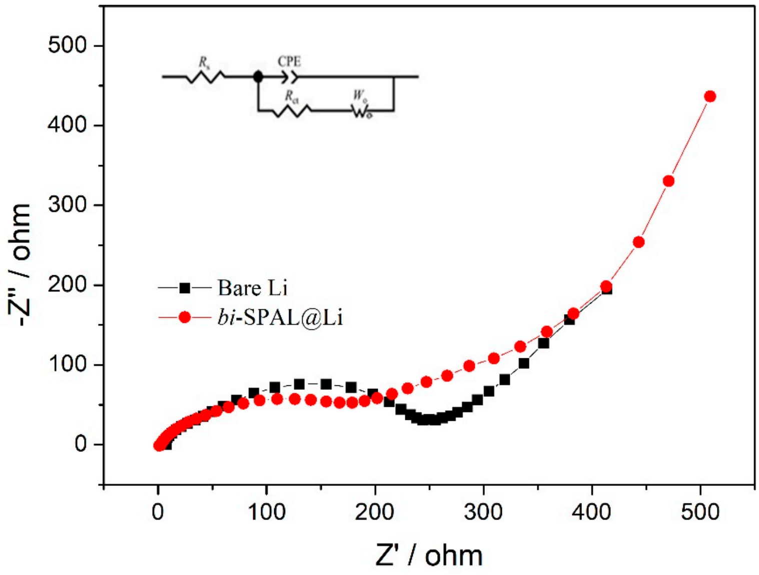

Figure S5. The Nyquist plots of the LFP|Li full cell after 20 cycles at 1 C with or without *bi*-SPAL.

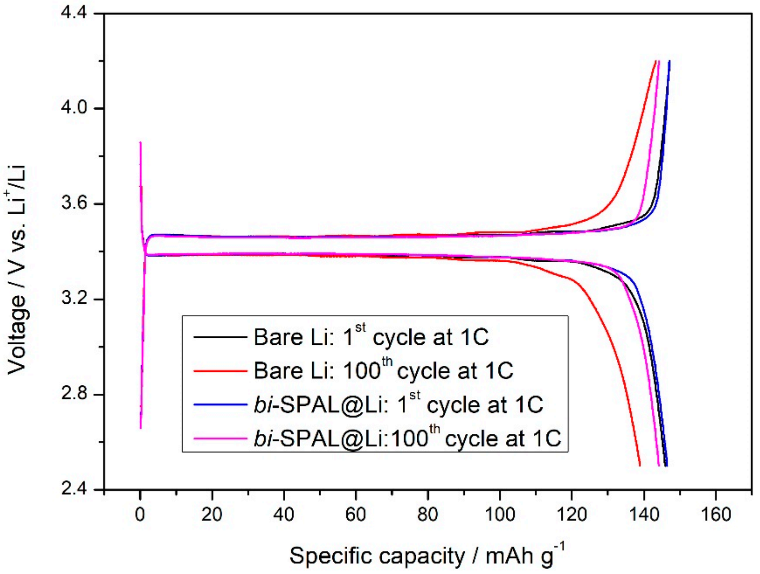

Figure S6. The charge/discharge curves of LFP/Li and LFP/*bi*-SPAL@Li cells at 1st cycle and 100th cycle.
